# Supplementary material for: An integrated transcriptomic and proteomic map of the mouse hippocampus at synaptic resolution
Source: Nat Commun. 2025 Aug 26;16:7942. doi: 10.1038/s41467-025-63119-5 (PMC12381143; doi:10.1038/s41467-025-63119-5)
Supplement: Supplementary file 1 — Supplementary Information [file 41467_2025_63119_MOESM1_ESM.pdf]

Supplementary figures for

An integrated transcriptomic and proteomic map of the mouse hippocampus at synaptic resolution

Eva Kaulich[1,#], Quinn Waselenchuk[1,2,#], Nicole Fürst[1], Kristina Desch[1], Janus Mosbacher[1], Elena Ciirdaeva[1], Marcel Juengling[1,3], Roshni Ray[1], Belquis Nassim-Assir[1], Georgi Tushev[1], Julian D. Langer[1,2], Erin M. Schuman[1,2,3*]

[#]These authors contributed equally: Eva Kaulich, Quinn Waselenchuk
*To whom correspondence should be addressed: erin.schuman@brain.mpg.de

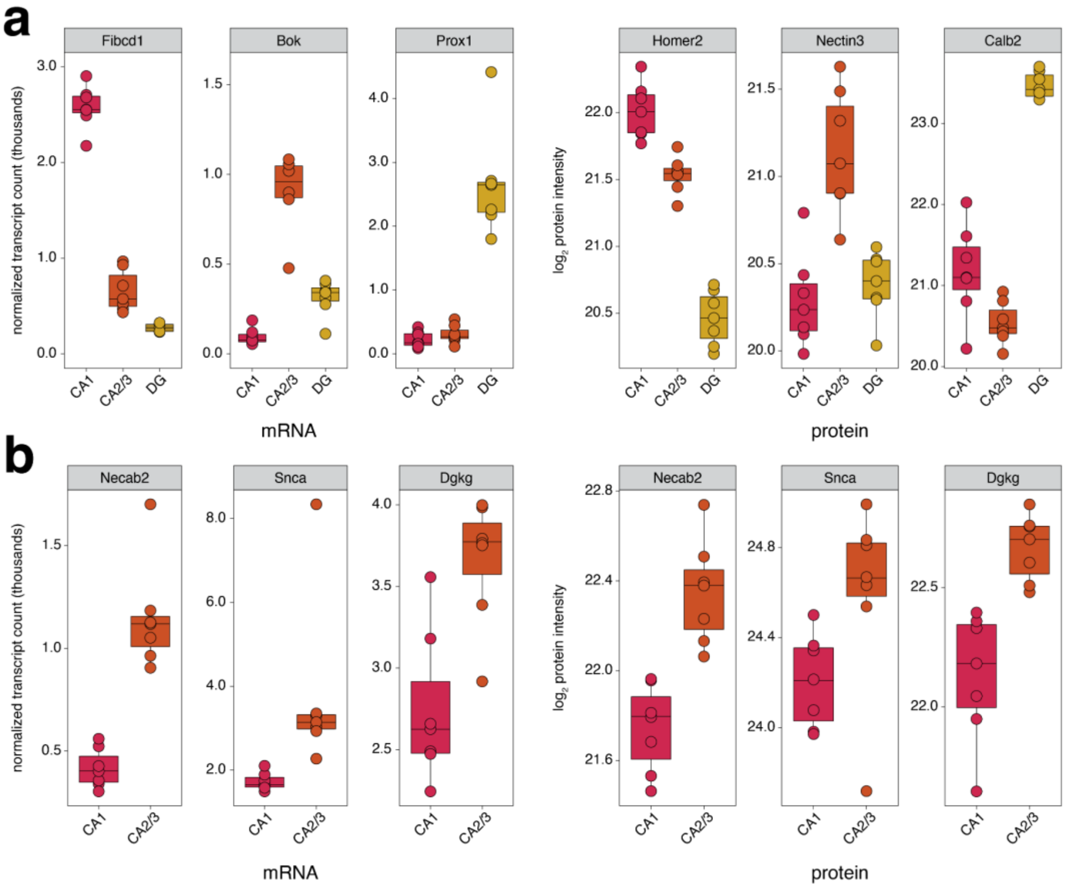

Supplementary Fig. 1: Detected enrichment of known molecular markers in hippocampal subregions. (a) Boxplots showing subregion-specific enrichment of previously described molecular markers at the mRNA (left) and protein (right) level, confirming the accuracy of subregion dissections. (b) Boxplots for mRNA (left) and protein (right) indicating known CA2 markers[1,2] are also enriched in the combined CA2/CA3 dissections in our dataset. Boxes indicate median (middle line) and interquartile ranges (IQR), with whiskers at 1.5 × IQR. Points represent values for each biological replicate (*n* = 7). Source data are provided as a source data file.

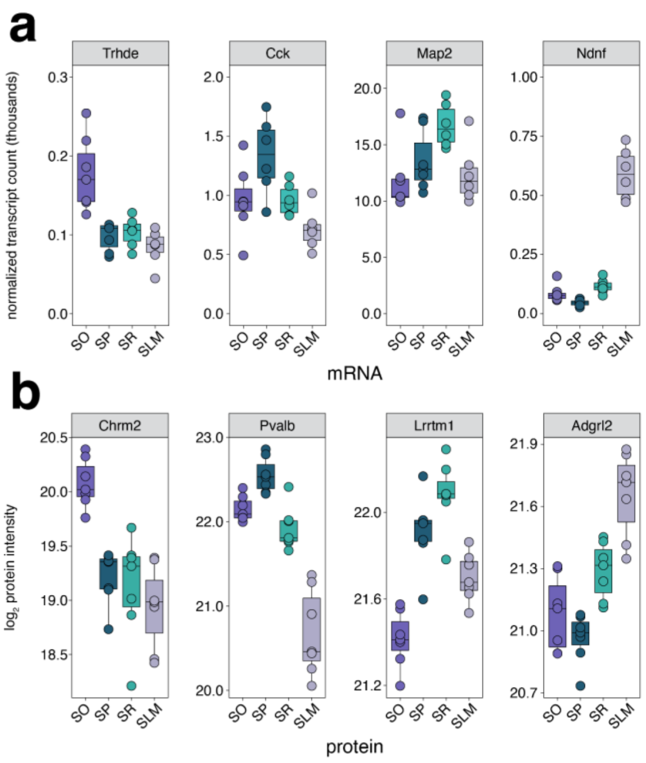

Supplementary Fig. 2: Detected enrichment of known molecular markers in CA1 strata. (a,b) Boxplots showing strata-specific enrichment of previously described molecular markers at the mRNA (a) and protein (b) level, confirming the accuracy of strata dissections. Boxes indicate median (middle line) and interquartile ranges (IQR), with whiskers at 1.5 × IQR. Points represent values for each biological replicate (n = 7). Source data are provided as a source data file.

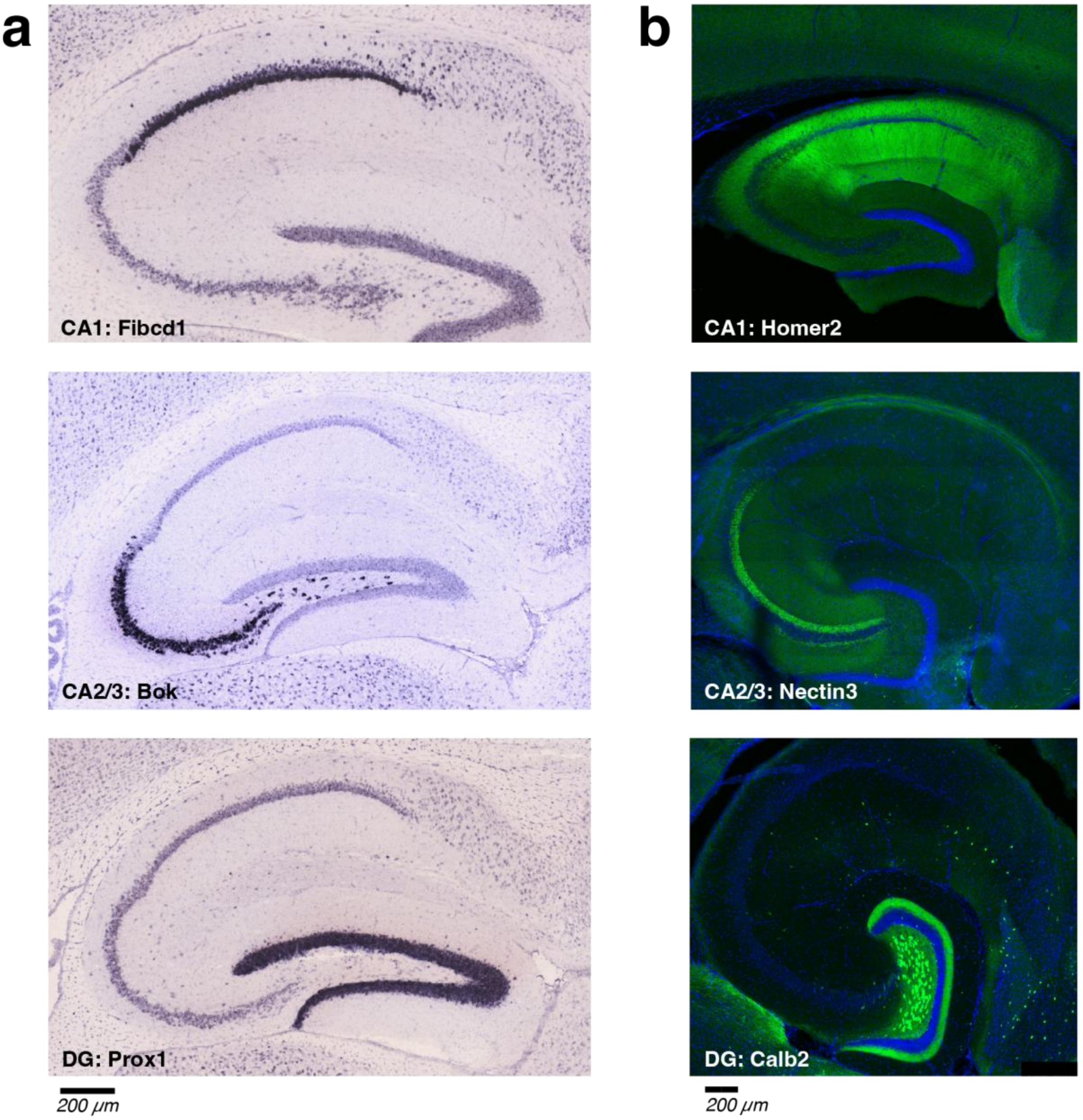

Supplementary Fig. 3: *In situ* hybridization (ISH) and immunofluorescence validation of hippocampal subregion-enriched transcripts and proteins. (a) ISH validation of transcripts that showed strong subregion-specific enrichment in our dataset. All images were obtained from the Allen Mouse Brain Atlas: *Fibcd1* (mouse.brain-map.org/gene/show/63140), *Bok* (mouse.brain-map.org/gene/show/31280), *Prox1* (mouse.brain-map.org/gene/show/18893) in the adult mouse brain. (b) Immunofluorescence validation of proteins that showed strong subregion-specific enrichment in our dataset: Homer2 in CA1, Nectin3 in CA2/3, and Calb2 in DG. DAPI fluorescence is depicted in blue and fluorescence of the respective protein is depicted in green.

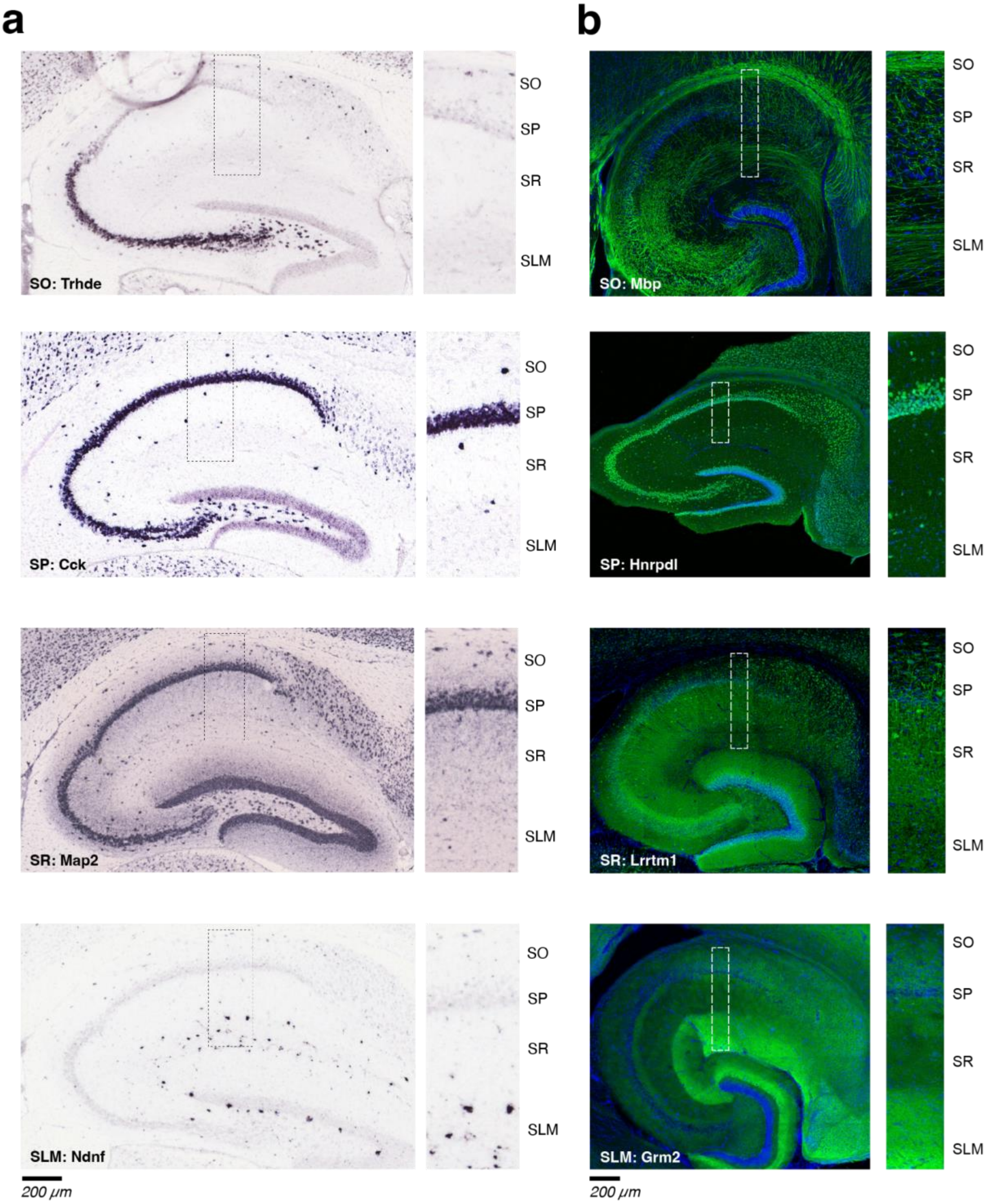

Supplementary Fig. 4: *In situ* hybridization (ISH) and immunofluorescence validation of CA1 strata-enriched transcripts and proteins. (a) ISH validation of transcripts that showed strong strata-specific enrichment in our dataset. All images were obtained from the Allen Mouse Brain Atlas: *Trhde* (mouse.brain-map.org/gene/show/79677195), *Cck* (mouse.brain-map.org/gene/show/12209), *Map2* (mouse.brain-map.org/gene/show/17523) and *Ndnf* (mouse.brain-map.org/gene/show/44012). (b) Immunofluorescence validation of proteins that showed strong strata-specific enrichment in our dataset: Mbp in *S. oriens*, Hnrpdl in *S.*

pyramidale, Lrrtm1 in *S. radiatum*, and Grm2 in *S. lacunosum-moleculare*. DAPI fluorescence is depicted in blue and fluorescence of the respective protein is depicted in green.

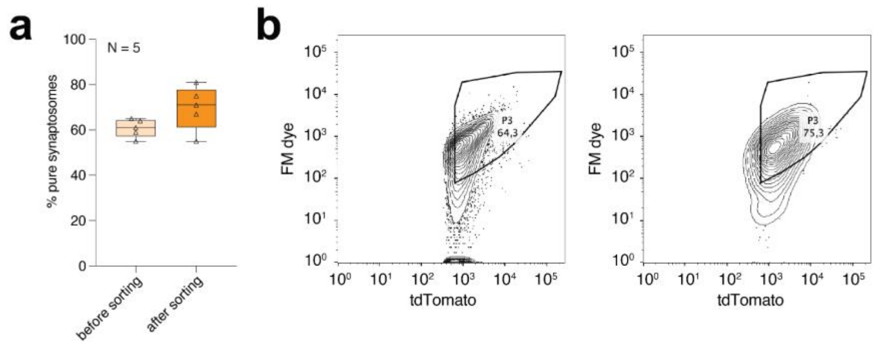

Supplementary Fig. 5: Purity, sorting efficiency, and gating strategy for Fluorescence Activated Synaptosome Sorting (FASS). (a) Plot indicating the relative abundance of each fluorescently labeled synaptosome type in the crude synaptosome fraction generated from each sample before and after sorting. Each triangle represents one replicate (*n* = 5). Boxes indicate median (middle line) and 25th to 75th percentiles. Error bars represent minima and maxima with all data points shown. (b) Gating strategy and example sorting efficiency. FASS contour plots (with outliers) showing the relative density of the targeted tdTomato-positive synaptic population in hippocampal synaptosomes prepared from Syn-1::SypTOM mice (64.3%; left). y axes represent fluorescence from a membrane dye (FM4-64) and x axes fluorescence from tdTomato. Following the initial sorting run (left), re-loading of the sorted synaptosomes indicated a high enrichment and purity (75.3%) of the Syn-1::SypTOM sample (right).

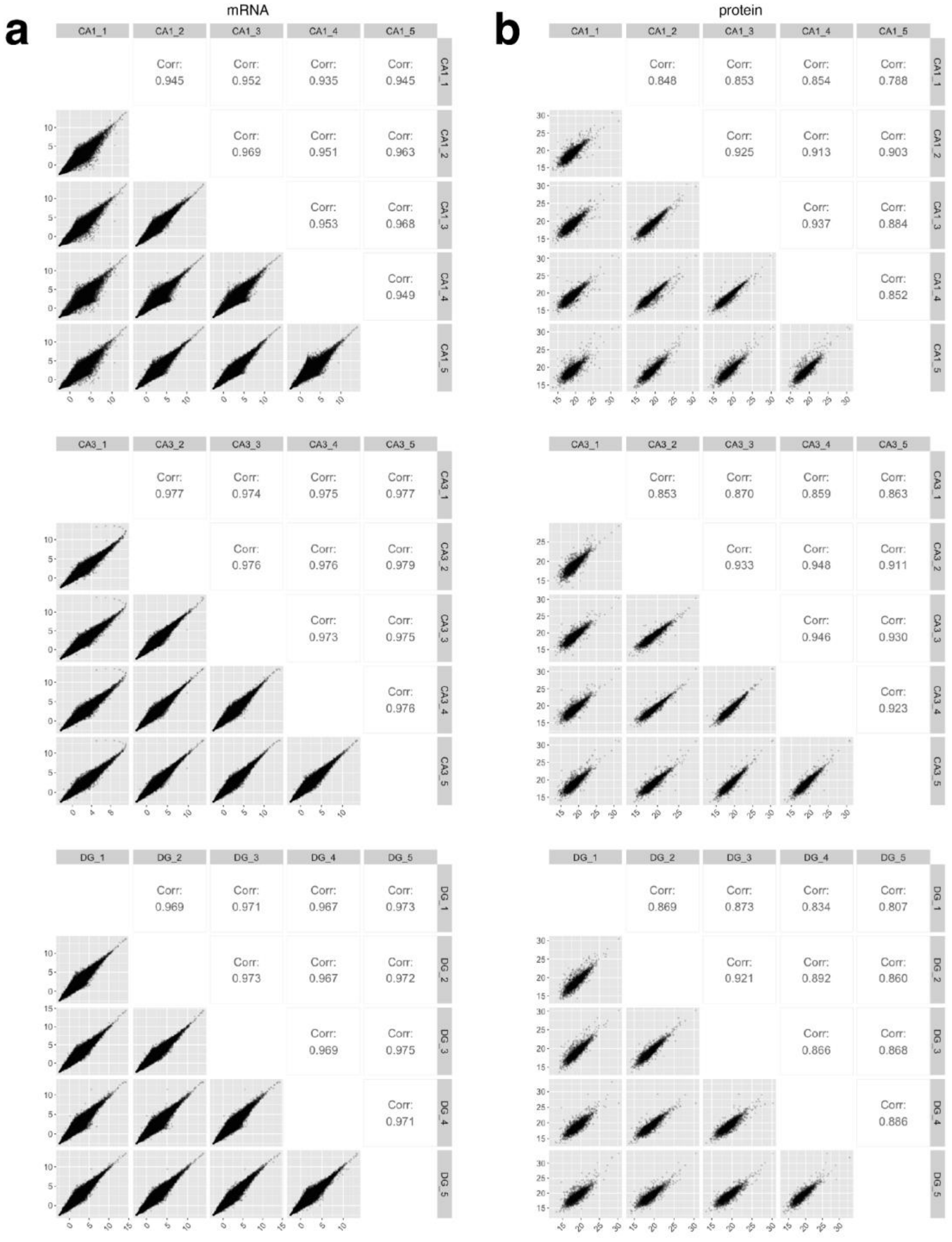

Supplementary Fig. 6: Subregion synaptosome replicates correlate strongly. Pairwise scatterplot matrices of (a) rlog-transformed counts and (b) log_2 LFQ protein intensities across replicates of synaptosomes isolated from hippocampal subregions (CA1, CA2/3, DG). Lower triangle panels show scatterplots between individual replicates. Upper triangle panels display correlation coefficients (Spearman for mRNA; Pearson for protein). Source data are provided as a source data file.

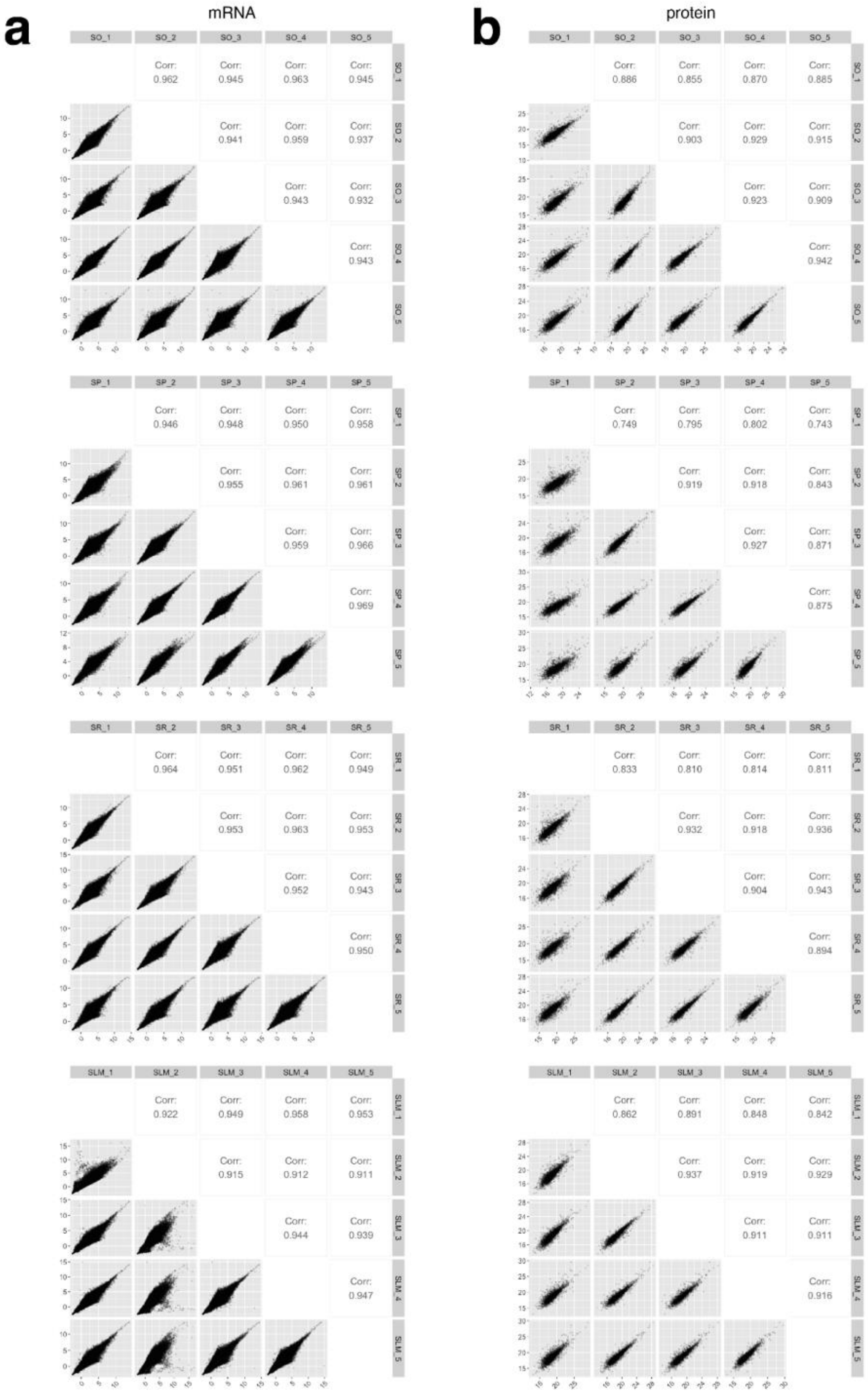

Supplementary Fig. 7: Strata synaptosome replicates correlate strongly. Pairwise scatterplot matrices of (a) rlog-transformed counts and (b) $\log_2$ LFQ protein intensities across replicates of synaptosomes isolated from CA1 strata (SO, SP, SR, SLM). Lower triangle panels show scatterplots between individual replicates. Upper triangle panels display correlation coefficients (Spearman for mRNA; Pearson for protein). Source data are provided as a source data file.

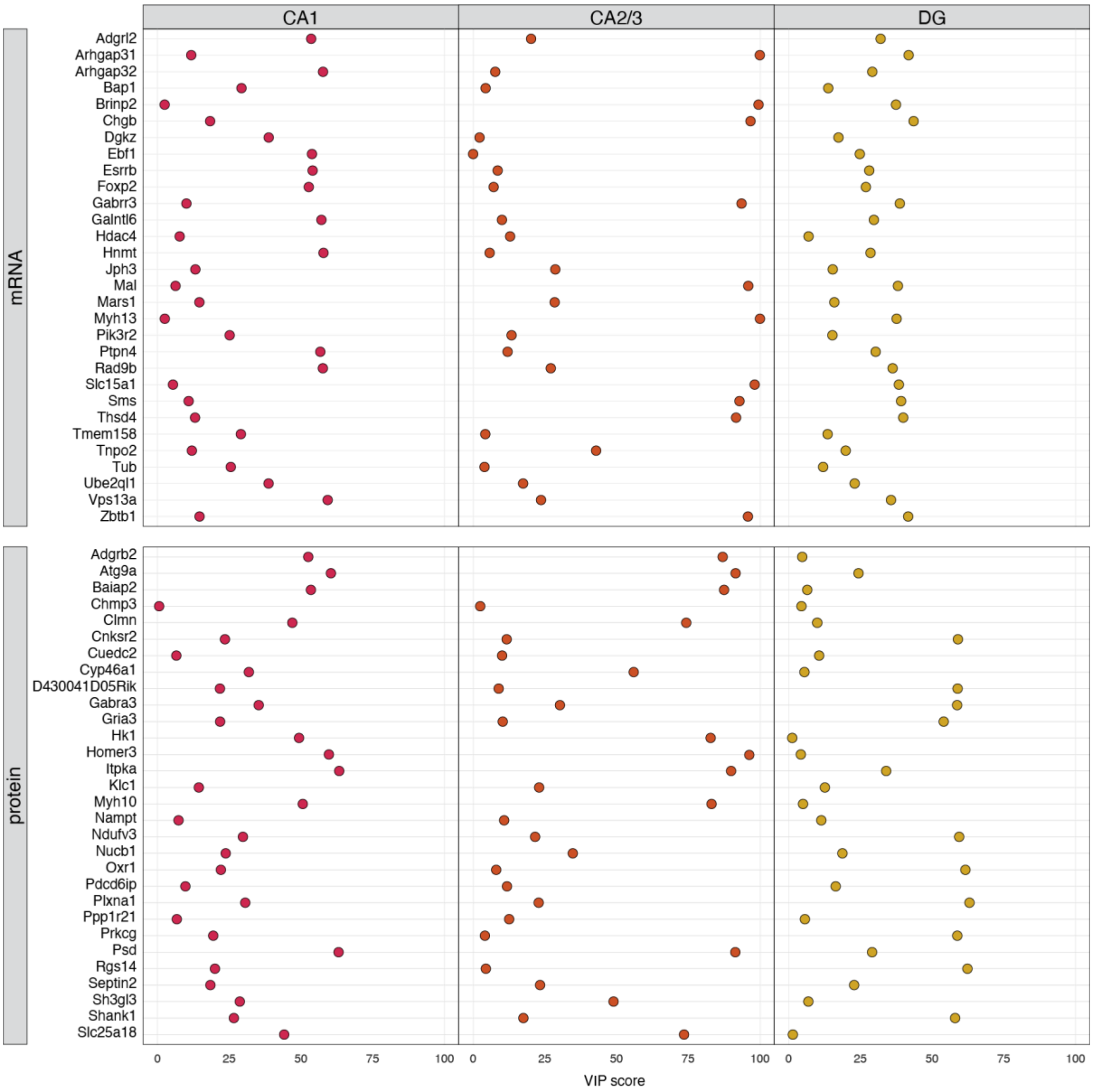

Supplementary Fig. 8: Loadings from PLS-DA analysis of subregion synaptic transcriptomes and proteomes. Variable Importance in Projection (VIP) plots showing VIP scores for the top ten loadings from the first three components in PLS-DA. VIP scores indicate the contribution of each variable to the discrimination between subregions in the PLS-DA model shown in Fig. 6d. Scores are scaled from 0-100. Higher VIP scores represent greater importance in distinguishing between subregions. Source data are provided as a source data file.

References

1. **Gerber, K. J. *et al.* Specific Proteomes of Hippocampal Regions CA2 and CA1 Reveal Proteins Linked to the Unique Physiology of Area CA2. *J. Proteome Res.* 18, 2571–2584 (2019).**
2. **Laham, B. J., Diethorn, E. J. & Gould, E. Newborn mice form lasting CA2-dependent memories of their mothers. *Cell Rep.* 34, 108668 (2021).**
