# Supplementary material for: An integrated transcriptomic and proteomic map of the mouse hippocampus at synaptic resolution
Source: Nat Commun. 2025 Aug 26;16:7942. doi: 10.1038/s41467-025-63119-5 (PMC12381143; doi:10.1038/s41467-025-63119-5)
Supplement: Supplementary file 2 — Description of Additional Supplementary Files [file 41467_2025_63119_MOESM2_ESM.pdf]

Description of Additional Supplementary Files

Supplementary Data 1: Output tables from transcriptomic analysis of tissue samples

Supplementary Data 2: Output tables from proteomic analysis of tissue and synaptosome samples

Supplementary Data 3: Integrated transcriptomic and proteomic analysis results

Supplementary Data 4: Output tables from transcriptomic analysis of synaptosome samples

Supplementary Data 5: List of primers and barcodes used for RNA-seq library preparation

Supplementary Data 6: Antibody information: supplier, catalogue numbers, and dilution factors
