# Supplementary material for: An integrated transcriptomic and proteomic map of the mouse hippocampus at synaptic resolution
Source: Nat Commun. 2025 Aug 26;16:7942. doi: 10.1038/s41467-025-63119-5 (PMC12381143; doi:10.1038/s41467-025-63119-5)
Supplement: Supplementary file 9 — Reporting Summary [file 41467_2025_63119_MOESM9_ESM.pdf]

nature portfolio

Corresponding author(s): Erin M Schuman

Last updated by author(s): Jul 21, 2025

Reporting Summary

Nature Portfolio wishes to improve the reproducibility of the work that we publish. This form provides structure for consistency and transparency in reporting. For further information on Nature Portfolio policies, see our Editorial Policies and the Editorial Policy Checklist.

Statistics

For all statistical analyses, confirm that the following items are present in the figure legend, table legend, main text, or Methods section.

n/a	Confirmed	
☐	☒	The exact sample size (*n*) for each experimental group/condition, given as a discrete number and unit of measurement
☐	☒	A statement on whether measurements were taken from distinct samples or whether the same sample was measured repeatedly
☐	☒	The statistical test(s) used AND whether they are one- or two-sided<br>*Only common tests should be described solely by name; describe more complex techniques in the Methods section.*
☐	☒	A description of all covariates tested
☐	☒	A description of any assumptions or corrections, such as tests of normality and adjustment for multiple comparisons
☐	☒	A full description of the statistical parameters including central tendency (e.g. means) or other basic estimates (e.g. regression coefficient) AND variation (e.g. standard deviation) or associated estimates of uncertainty (e.g. confidence intervals)
☐	☒	For null hypothesis testing, the test statistic (e.g. *F*, *t*, *r*) with confidence intervals, effect sizes, degrees of freedom and *P* value noted<br>*Give P values as exact values whenever suitable.*
☐	☒	For Bayesian analysis, information on the choice of priors and Markov chain Monte Carlo settings
☐	☐	For hierarchical and complex designs, identification of the appropriate level for tests and full reporting of outcomes
☐	☒	Estimates of effect sizes (e.g. Cohen's *d*, Pearson's *r*), indicating how they were calculated

Our web collection on statistics for biologists contains articles on many of the points above.

Software and code

Policy information about availability of computer code

Data collection	Illumina platform (Illumina NextSeq2000) was used for RNAseq
Data analysis	Open-source R packages were used to analyse transcriptomics and proteomics data (DESeq2 v1.46.0, MS-DAP, ClusterProfiler v4.14.6, ggplot2 v3.5.2, pheatmap v1.0.13, apeglm v1.28.0, caret v7.0.1, pls v2.8.5, dplyr v1.1.4, stringr v1.5.1, DOSE v4.0.1), as well as the open-source software DIA-NN (v1.8.2 beta 27) and the DIAgui Shiny app.

For manuscripts utilizing custom algorithms or software that are central to the research but not yet described in published literature, software must be made available to editors and reviewers. We strongly encourage code deposition in a community repository (e.g. GitHub). See the Nature Portfolio guidelines for submitting code & software for further information.

Data

Policy information about availability of data

All manuscripts must include a data availability statement. This statement should provide the following information, where applicable:

- Accession codes, unique identifiers, or web links for publicly available datasets
- A description of any restrictions on data availability
- For clinical datasets or third party data, please ensure that the statement adheres to our policy

The raw RNA-sequencing data for tissue and synaptosomes are available at the NCBI Sequence Read Archive (SRA) under the reference number: PRJNA1142450 ("RNAseq of microdissected mouse hippocampal subregions and CA1 strata") and PRJNA1142761 ("RNA-Seq of sorted synaptosomes from microdissection of mouse

hippocampal subregions and CA1 strata") and available upon reviewer request. Mass spectrometry data associated with this manuscript are being uploaded to the PRIDE repository (PXD054879). mRNA and proteins can also be assessed at our lab's public interface https://syndive.org/. All customized R scripts have been deposited on GitLab and are accessible via the following DOI: https://doi.org/10.17617/1.1WVD-V337.

Research involving human participants, their data, or biological material

Policy information about studies with human participants or human data. See also policy information about sex, gender (identity/presentation), and sexual orientation and race, ethnicity and racism.

Reporting on sex and gender	*Use the terms sex (biological attribute) and gender (shaped by social and cultural circumstances) carefully in order to avoid confusing both terms. Indicate if findings apply to only one sex or gender; describe whether sex and gender were considered in study design; whether sex and/or gender was determined based on self-reporting or assigned and methods used. Provide in the source data disaggregated sex and gender data, where this information has been collected, and if consent has been obtained for sharing of individual-level data; provide overall numbers in this Reporting Summary. Please state if this information has not been collected. Report sex- and gender-based analyses where performed, justify reasons for lack of sex- and gender-based analysis.*
Reporting on race, ethnicity, or other socially relevant groupings	*Please specify the socially constructed or socially relevant categorization variable(s) used in your manuscript and explain why they were used. Please note that such variables should not be used as proxies for other socially constructed/relevant variables (for example, race or ethnicity should not be used as a proxy for socioeconomic status). Provide clear definitions of the relevant terms used, how they were provided (by the participants/respondents, the researchers, or third parties), and the method(s) used to classify people into the different categories (e.g. self-report, census or administrative data, social media data, etc.) Please provide details about how you controlled for confounding variables in your analyses.*
Population characteristics	*Describe the covariate-relevant population characteristics of the human research participants (e.g. age, genotypic information, past and current diagnosis and treatment categories). If you filled out the behavioural & social sciences study design questions and have nothing to add here, write "See above."*
Recruitment	*Describe how participants were recruited. Outline any potential self-selection bias or other biases that may be present and how these are likely to impact results.*
Ethics oversight	*Identify the organization(s) that approved the study protocol.*

Note that full information on the approval of the study protocol must also be provided in the manuscript.

Field-specific reporting

Please select the one below that is the best fit for your research. If you are not sure, read the appropriate sections before making your selection.

☒ Life sciences ☐ Behavioural & social sciences ☐ Ecological, evolutionary & environmental sciences

For a reference copy of the document with all sections, see nature.com/documents/nr-reporting-summary-flat.pdf

Life sciences study design

All studies must disclose on these points even when the disclosure is negative.

Sample size	Sample size were used based on prior research in the field
Data exclusions	We excluded datapoints that did not pass initial quality control after RNA-sequencing based on low counts and therefore PCA outliers
Replication	We collected 7 replicates for the tissue data and 5 replicates for the synaptosome data and used PCA to confirm reproducibility
Randomization	We randomlys split the sample for downstream RNAseq and LC-MS/MS
Blinding	Blinding was not applicable as the genotypes and samples come from the same mouse and no experimental manipulations were used

Reporting for specific materials, systems and methods

We require information from authors about some types of materials, experimental systems and methods used in many studies. Here, indicate whether each material, system or method listed is relevant to your study. If you are not sure if a list item applies to your research, read the appropriate section before selecting a response.

Materials & experimental systems

n/a	Involved in the study
☒	☐ Antibodies
☒	☐ Eukaryotic cell lines
☒	☐ Palaeontology and archaeology
☐	☒ Animals and other organisms
☒	☐ Clinical data
☒	☐ Dual use research of concern
☒	☐ Plants

Methods

n/a	Involved in the study
☒	☐ ChIP-seq
☐	☒ Flow cytometry
☒	☐ MRI-based neuroimaging

Animals and other research organisms

Policy information about studies involving animals; ARRIVE guidelines recommended for reporting animal research, and Sex and Gender in Research

Laboratory animals	For this study we used Cre-positive and Cre-negative F1 littermates from offspring of a cross between hemizygous Syn1-Cre female mice (B6.Cg-Tg(Syn1-cre)671Jxm/J, RRID:IMSR_JAX:003966) and homozygous condSypTom males (B6;129S-Gt(ROSA)26Sortm34.1(CAG-Syp/tdTomato)Hze/J obtained from The Jackson Laboratory, RRID:IMSR_JAX:012570)1
Wild animals	study did not involve wild animals
Reporting on sex	One male and one female mouse was pooled together for each replicate, sex was not considered as a variable, findings apply to both sexes
Field-collected samples	study did not involve field-collected samples
Ethics oversight	The procedures involving animal treatment and care were conducted in conformity with the institutional guidelines that are in compliance with national and international laws and policies (DIRECTIVE 2010/63/EU; German animal welfare law; FELASA guidelines). The animals were euthanized according to annex 2 of § 2 Abs. 2 Tierschutz-Versuchstier-Verordnung and animal numbers were reported to the local authorities (Regierungspräsidium Darmstadt).

Note that full information on the approval of the study protocol must also be provided in the manuscript.

Plants

Seed stocks	*Report on the source of all seed stocks or other plant material used. If applicable, state the seed stock centre and catalogue number. If plant specimens were collected from the field, describe the collection location, date and sampling procedures.*
Novel plant genotypes	*Describe the methods by which all novel plant genotypes were produced. This includes those generated by transgenic approaches, gene editing, chemical/radiation-based mutagenesis and hybridization. For transgenic lines, describe the transformation method, the number of independent lines analyzed and the generation upon which experiments were performed. For gene-edited lines, describe the editor used, the endogenous sequence targeted for editing, the targeting guide RNA sequence (if applicable) and how the editor was applied.*
Authentication	*Describe any authentication procedures for each seed stock used or novel genotype generated. Describe any experiments used to assess the effect of a mutation and, where applicable, how potential secondary effects (e.g. second site T-DNA insertions, mosiacism, off-target gene editing) were examined.*

Flow Cytometry

Plots

Confirm that:

☒ The axis labels state the marker and fluorochrome used (e.g. CD4-FITC).

☒ The axis scales are clearly visible. Include numbers along axes only for bottom left plot of group (a 'group' is an analysis of identical markers).

☒ All plots are contour plots with outliers or pseudocolor plots.

☒ A numerical value for number of cells or percentage (with statistics) is provided.

Methodology

Sample preparation	Percoll-succrose gradient crude synaptosome preparation (Westmark et al., 2011, doi:10.3791/3196)
Instrument	FACSAria Fusion (BD Biosciences)

Software	FACSDiva, FlowJo, Flow Cytometry was only used for sample collection
Cell population abundance	Synaptosomes, approximately 60% to almost 80% purity
Gating strategy	We used a previously generated gates and settings (van Oostrum et al., 2023 (https://doi.org/10.1016/j.cell.2023.09.028) referred to as P3 and P2. P3 to obtain the synaptosome population that selects particles that are double-positive for tdTomato and membrane dye FM4-64 (sorted synaptosomes) by thresholding against synaptosomes from wild-type mice (van Oostrum et al., 2023). For control synaptosomes (P2) all non-doublet particles above the FM4-64 threshold were sorted (P2). For each sorted sample (P3) as well as the matching control sample (P2).

☒ Tick this box to confirm that a figure exemplifying the gating strategy is provided in the Supplementary Information.
